# Active Compounds of Rhubarb Root and Rhizome in Animal Model Experiments of Focal Cerebral Ischemia

**DOI:** 10.1155/2015/210546

**Published:** 2015-10-01

**Authors:** Ai-ju Liu, Liang Song, Yan Li, Xiao-guang Zhang, Zi-xian Chen, Li-bo Huang, Hong-feng Zhang, Guo-qing Zheng

**Affiliations:** Department of Neurology, The Second Affiliated Hospital & Yuying Children's Hospital of Wenzhou Medical University, Wenzhou 325027, China

## Abstract

Rhubarb root and rhizome (RRR) has been clinically used for stroke at least 2000 years and is still used in modern times in both China and elsewhere worldwide. The objective of present study was to evaluate the efficacy of active compounds of RRR (ACRRR) for experimental ischemic stroke. Studies of ACRRR in animal models of ischemic stroke were identified from 5 databases until April 2014. Study quality for each included article was evaluated according to the CAMARADES 10-item checklist. Outcome measures were neurological deficit score and infarct size. All the data were analyzed using RevMan 5.1 software. As a result, 20 studies were identified describing procedures involving 577 animals. The quality score of studies ranges from 2 to 6, and the median was 3.4. Six studies showed significant effects of ACRRR for improving infarct size compared with model group (*P* < 0.01). Six studies indicated significant effects of ACRRR for improving the neurological deficit scores according to Zea longa criterion or eight-point criterion (*P* < 0.01). In conclusion, these findings demonstrated a possible efficacy of ACRRR that have potential neuroprotective effect for experimental ischemic stroke. However, these apparently positive findings should be interpreted with caution because of the methodological flaws.

## 1. Introduction

Stroke is a major cause of disability and the second most common cause of death worldwide [1]. The burden of stroke will increase greatly during the next 20 years because of the aging population, especially in developing countries [2] such as in China where stroke has already become the leading cause of death [3]. Ischemic stroke is the most common type of stroke, accounting for almost 80% of all types of strokes. Unfortunately, intravenously recombinant tissue plasminogen activator (rtPA) is so far the only approved thrombolytic by Food and Drug Administration for treating ischemic stroke within 4.5 hours of stroke onset [4]. However, rtPA remains largely underutilized because of the short therapeutic window and the incidence of intracranial hemorrhages [5]. Owing to the limitations of the current available treatments, complementary and/or alternative medicine (CAM) is thus increasingly sought to treat stroke worldwide.

Traditional Chinese Medicine (TCM), as a form of CAM, has been used in stroke patients for thousands of years and is still being commonly used in modern times in both China and elsewhere worldwide [6]. In TCM treatment of stroke, the rhubarb root and rhizome (RRR) and RRR-based Chinese herbal prescriptions, known as Tongfu method in TCM theory, were one of the essential methods for acute stroke [7]. RRR, Dahuang in Chinese name and Radix et Rhizoma Rhei in Latin name, can purge accumulation, cool blood, drain damp-heat, and invigorate blood according to TCM theory. RRR has been clinically used for a long history of 2000 years [8], which was documented in the earliest complete Pharmacopoeia of China,* Shennongbencaojing* (*Shennong's Classic of Materia Medica*) at the Warring States Period to the Han Dynasty (221 BC-220 AD). The use of RRR in treatment of stroke can be traced back to the Eastern Han Dynasty (206 BC-220 AD). Doctor Zhang Zhongjing (AD152-219), one of the most eminent Chinese physicians, has first applied RRR as one of the principal herbs in Fengyin Decoction to treat poststroke epilepsy patients induced evil-wind due to excessive heat [9]. In modern time, RRR is still being used to treat stroke and often present as a principle drug in Chinese herbal prescriptions for the treatment of stroke. In our group, we have conducted a systematic review assessing the effects of RRR-based prescriptions on patients suffering from acute ischemic stroke; the results indicated that this area is worthy of improvement and development for further research [7].

In the current Chinese Pharmacopoeia, RRR is listed as the dry root and rhizome of* Rheum officinale* Baill.,* Rheum palmatum* L., and* Rheum tanguticum* Maxim. The extensive phytochemical research on RRR has isolated and identified about 200 chemical compounds [8], such as anthraquinones, dianthrones, stilbenes, anthocyanins, flavonoids, tannins, organic acids, and chromones [10]. Neuroprotection refers to the concept of applying a therapy that directly affects the brain tissue to salvage or delay the infarction of the still-viable ischemic penumbra, rather than reperfusing the tissue [4]. Pharmacological agents targeted the harmful molecular events that contribute to acute ischemic injury pathophysiology, including glutamate release, glutamate receptor activation, excitotoxicity, Ca^2+^ influx into cells, mitochondrial dysfunction, activation of many intracellular enzymes, free radical production, nitric oxide production, inflammation, necrosis, and apoptosis [11]. For example, the registered neuroprotective agents (Internet Stroke Center, 2011) included calcium channel blocker, calcium chelator, free radical scavenger/antioxidant, gamma aminobutyric acid (GABA) agonist, glutamate antagonist, growth factor, leukocyte adhesion inhibitor, nitric oxide inhibitor, opioid antagonist, phosphatidylcholine precursor, serotonin agonist, sodium channel blocker, potassium channel opener, and mechanism unknown or uncertain [12]. Although at least 26 phase 2 and 3 trials of neuroprotectants have completed since 2000, no definite pharmacological agents can limit the cellular effects of acute ischemia or reperfusion that demonstrate safety and efficacy after stroke in clinical studies [13]. Over the past decades, growing evidence indicates that the active compounds of RRR (ACRRR), including rhubarb aglycone (the five components including aloe-emodin, rhein, emodin, chrysophanol, and physcion), rhubarb glycosides (anthraquinone glycosides and double anthrone glycoside), chrysophanol, chrysophanol liposome, emodin, aloe-emodin, physcion, and rhein are responsible for the main pharmacological effects on the stroke and exert potentially neuroprotective function against cerebral ischemic injury [14–33]. The use of systematic review in the preclinical assessment of candidate neuroprotectants can more systematically assess the efficacy, identify an area for testing in further animal experiments, and provide robust information about the characteristics of individual drugs and the basis for a new classification of neuroprotective drugs [34]. In addition, systematic reviews of preclinical data can inform the planning and improve the likelihood of success of future clinical trials [35]. We thus conducted a preclinical systematic review to evaluate ACRRR for experimental ischemic stroke.

## 2. Methods

### 2.1. Database and Literature Search Strategies

We identified studies of ACRRR in animal models of ischemic stroke from PubMed, EMBASE, Chinese National Knowledge Infrastructure (CNKI), VIP information database, and Wanfang data Information Site. All of the searches were performed until April 2014. The search term used was (“ACRRR” OR “Rhubarb aglucone” OR “Rhubarb glycosides” OR “Chrysophanol” OR “Chrysophanol liposome” OR “Emodin” OR “Aloe-emodin” OR “Physcion” OR “Rhein”) AND [“isch(a)emic stroke” OR “cerebral infarct” OR “middle carotid artery occlusion (MCAO)” OR “cerebral isch(a)emica/reperfution”]. All searches were limited to studies on animals. We also manually searched published abstracts of scientific meetings and asked senior authors of identified publications for references of other studies.

### 2.2. Inclusion Criteria

We included studies of the effect of ACRRR in animal models of focal cerebral ischemia, in which the outcome was measured as neurological function score (NFS) and (or) infarct size/infarct volume. To prevent bias, inclusion criteria were prespecified as follows: (1) experimental ischemic stroke was induced by temporary MCAO or permanent MCAO; (2) ACRRR referred to any chemical compounds of RRR; (3) infarct size/infarct volume and (or) NFS were compared with control animals receiving vehicle or no treatment. Prespecified exclusion criteria were treatment with single RRR or RRR-based prescriptions, nonfocal cerebral ischemia model, no control group, and duplicate publications.

### 2.3. Data Extraction

Two authors independently screened abstracts, and the resulting manuscripts were approved by corresponding author (Guo-qing Zheng). The following information was extracted from the complete manuscripts of the qualified studies: (1) publication year and the first author's name, model of ischemic stroke (transient or permanent); (2) the characteristics of animals used including animal number, species, sex, weight, age, and any comorbidity; (3) the information of treatment used in experimental group including the types of ACRRR, method of administration, and duration of treatment; (4) outcome measures and timing for outcomes assessments also included infarct size/infarct volume and (or) NFS were especially extracted separately. If outcomes were performed at different time points, only the final test was included. If the experimental group of animals received various doses of the drug therapy, only the data of highest dose of the drug was included. If the experimental group of animals received more than one kind of effective component of RRR intervention, the data of every intervention was included. If published data were incomplete, we contacted authors to obtain further information. For each comparison, we extracted data of mean value and standard deviation from each experimental and control group of every study.

### 2.4. Quality Assessment

We evaluated the methodological quality of the included studies using the collaborative approach to meta-analysis and review of animal data in experimental stroke (CAMARADES) 10-item quality checklist [34]. One point was awarded for each of (1) publication in a peer-reviewed journal; (2) statement of temperature control; (3) random allocation to groups; (4) allocation concealment; (5) blinded assessment of outcome; (6) use of anesthetic without significant intrinsic neuroprotective activity; (7) appropriate animal model (aged, diabetic, or hypertensive); (8) sample size calculation; (9) compliance with animal welfare regulations; (10) statement of potential conflict of interests. Two authors independently assessed study quality and any disagreements were solved through discussion or consultation with corresponding author (Guo-qing Zheng).

### 2.5. Statistical Analysis

All IS and NFS were considered as continuous data, and then an estimate of the combined effect sizes utilizing standard mean difference (SMD) with the random effects model was given. In the present meta-analysis, the results using the random effects model were presented because heterogeneity between multistudies has to be taken into account. *I* ^2^ statistic was used to assess heterogeneity. The significance of differences between *n* groups was assessed by partitioning heterogeneity and by using the *χ* ^2^ distribution with *n* − 1 degrees of freedom (df), where *n* equals the number of groups. Publication bias was assessed using a funnel plot. Probability values 0.05 were considered significant. All analyses were performed with Revman version 5.1 provided by the Cochrane Collaboration.

## 3. Results

### 3.1. Study Inclusion

**Figure 1:**
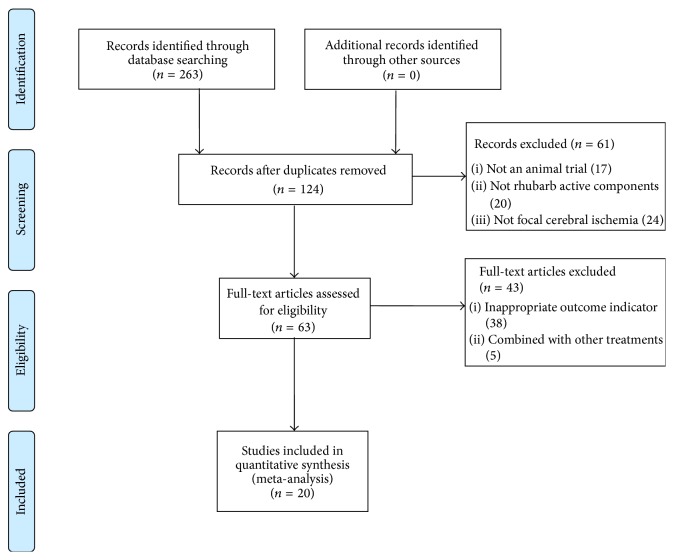
PRISMA 2009 flow diagram.

We identified 263 potentially relevant articles, and 139 were excluded because they were duplicates. Through screening titles and abstracts, 61 papers were excluded with at least one of following reasons: (1) not an animal research; (2) not ACRRR intervention; (3) not a research about stroke or ischemic stroke. By reading the full text of the remaining 63 articles, 38 were excluded because the outcome measure was neither NFS nor infarct size/infarct volume; 5 were excluded because of combination with other treatments. Ultimately, 20 eligible studies were identified [14–33]. The screening process is summarized in a flow diagram (Figure 1).

### 3.2. Study Characteristics

A total of 577 subjects were included in the 20 studies, of whom 282 were in the experimental group and 295 were in the control group. Two studies [15, 33] were published in English and eighteen studies [14, 16–32] were published in Chinese between 2004 and 2015. Seventeen studies [14, 16–31] used male/female Sprague-Dawley rat models; 1 study [15] used male Wistar rats; 1 study [32] used male Kunming mice models; 1 study [33] used male CD1 mice model. Among 20 included studies, 12 studies [14–16, 18–24, 27, 28] used permanent MCAO models; 6 studies [25, 26, 29–31, 33] used temporary MCAO models; 1 study [17] used embolic MCAO models; the remaining 1 study [32] used Himori [36] method to induce mice models of cerebral ischemia/reperfusion. All 20 studies reported NFS, and 14 studies [14–17, 19, 20, 22, 25, 27, 28, 30, 31, 33] reported IS. However, there were three neurological grading systems which were used to measure NFS in 20 studies. Eight studies [14, 15, 25, 26, 29–31, 33] used Zea longa criterion [37]; eleven studies [16–24, 27, 28] adopt eight-point criterion [38]; the remaining one study [32] used Garcia criterion [39]. Four studies [25, 29, 32, 33] used anesthesia to execute the animals, whereas the rest of studies did not report the method of executing the animals. Eleven studies [14, 16, 18–20, 22–24, 26–28] used random digits table to generate experimental and control groups, whereas the rest of studies did not mention the random method, which only reported random allocation to groups. The characteristics of the 20 included studies were summarized in detail in Table 1.

### 3.3. Study Quality

All studies were publications in a peer-reviewed journal. Fourteen studies [15, 19, 20, 22, 23, 26–29, 32, 33] reported control of temperature, including control of the room and rats anal temperature. All studies described random allocation to groups, of which 11 studies used random number table method [14, 16, 18–20, 22–24, 26–28]. Masked assessment of outcome was used in 1 study [16]. Chloral hydrate was used as anesthetic in 8 studies [14–16, 25, 29, 30, 32, 33]; pentobarbital was used in 1 study [26], while there was no report of anesthetics in the remaining 11 studies. Six studies described a sample size calculation [16, 17, 20, 22, 26, 32]. One study [33] reported a compliance with animal welfare regulations or mentioned a statement of potential conflict of interests. None of studies described masked induction of ischemia and appropriate animal models (aged, diabetic, or hypertensive). The quality score of studies ranges from 2 to 6, and the median was 3.4. The methodological quality of each study was summarized in Table 2.

### 3.4. Effectiveness

#### 3.4.1. Infarct Size/Infarct Volume

**Figure 2:**
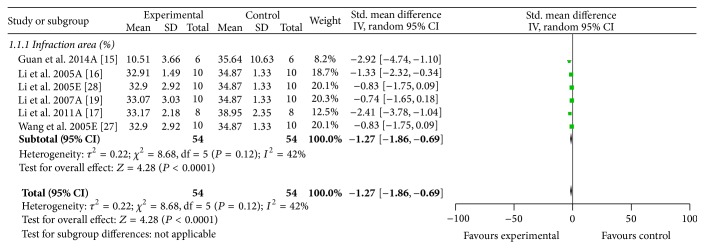
The forest plot: effects of active compounds of rhubarb root and rhizome for improving infarct size compared with middle carotid artery occlusion group.

Fourteen studies [14–17, 19, 20, 22, 25, 27, 28, 30, 31, 33] used infarct size/infarct volume as primary outcome measures. Meta-analysis of seven studies [15–17, 19, 27, 28, 33] showed significant effects of ACRRR for improving infarct size compared with MCAO group (*n* = 120, SMD −1.60, 95% CI: −2.48 ~ −0.72, *P* = 0.0004; heterogeneity *χ* ^2^ = 21.06, *P* = 0.002, *I* ^2^ = 72%). We used sensitivity analyses omitting one study at a time from the original analysis. One study [33] reported that the included animals were mice, while other six studies used rats. Thus, this study was considered as the potential sources of the heterogeneity. Meta-analysis of six studies [15–17, 19, 27, 28] indicated that the animal species may be the explanation for the heterogeneity. Six studies indicated that ACRRR significantly improved infarct size compared with MCAO group (*n* = 108, SMD −1.27, 95% CI: −1.86 ~ −0.69, *P* < 0.0001; heterogeneity *χ* ^2^ = 8.68, df = 5, *P* = 0.12, *I* ^2^ = 42%, Figure 2). The remaining seven studies [14, 20, 22, 25, 29–31] failed to pool analysis due to data demonstrated in the form of infarct volume or the absence of primary data, but all of them reported the significant effects of ACRRR for reducing the infarct size/infarct volume compared with the control group (*P* < 0.05 or *P* < 0.01).

#### 3.4.2. NFS

**Figure 3:**
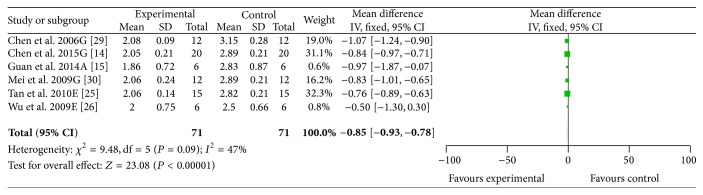
The forest plot: effects of active compounds of rhubarb root and rhizome for improving the neurological function score according to Zea longa criterion compared with middle carotid artery occlusion group.

**Figure 4:**
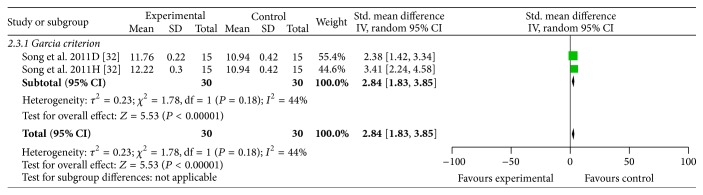
The forest plot: effects of active compounds of rhubarb root and rhizome for improving the neurological function score according to Garcia criterion compared with middle carotid artery occlusion group.

**Figure 5:**
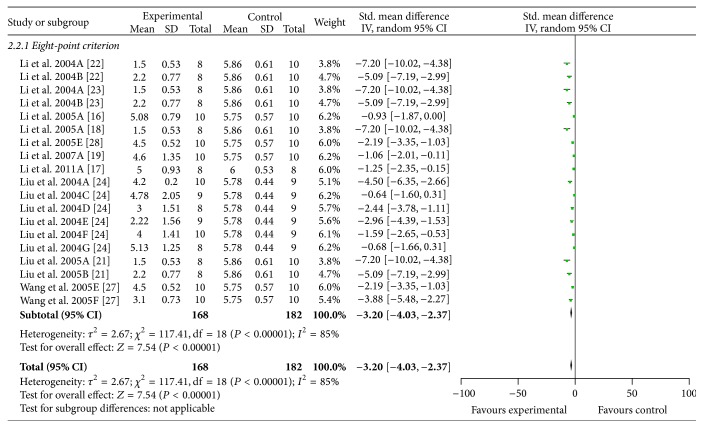
The forest plot: effects of active compounds of rhubarb root and rhizome for improving the neurological function score according to eight-point criterion compared with middle carotid artery occlusion group.

**Figure 6:**
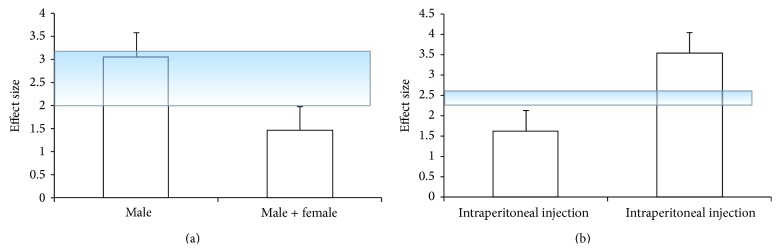
Subgroup analysis: (a) point estimates of effect size and 95% CIs by animal species; (b) point estimates of effect size and 95% CIs by route of drug delivery.

Based on the different neurological grading systems, eight studies [14, 15, 25, 26, 29–31, 33] used Zea longa criterion as measuring method of NFS. Meta-analysis of six studies [14, 15, 25, 26, 29, 30] indicated significant effects of ACRRR for improving the NFS according to Zea longa criterion (*n* = 142, SMD −0.85, 95% CI: −0.93 ~ −0.78, *P* < 0.00001; heterogeneity *χ* ^2^ = 9.48, df = 5, *P* = 0.09, *I* ^2^ = 47%, Figure 3) compared with the control group. Two studies [31, 33] also showed the significant effects of ACRRR for reducing NFS according to Zea longa criterion compared with the control group (*P* < 0.01) but failed to pool analysis due to the absence of primary data. One study [32] used Garcia criterion as measuring method of NFS. Meta-analysis of two comparisons of this study [32] showed significant effects of ACRRR for improving the NFS according to Garcia criterion compared with control group (*n* = 60, SMD 2.84, 95% CI: 1.83~3.85, *P* < 0.00001; heterogeneity *χ* ^2^ = 1.78, *P* = 0.18, *I* ^2^ = 44%, Figure 4). Eleven studies [16–24, 27, 28] used eight-point criterion as measuring method of NFS. Ten studies [16–19, 21–24, 27, 28] indicated that NFS was significantly improved in ACRRR group compared with control group according to eight-point criterion (*n* = 350, SMD −3.20, 95% CI: −4.03 ~ −2.37, *P* < 0.00001; heterogeneity *χ* ^2^ = 117.41, *P* < 0.00001, *I* ^2^ = 85%, Figure 5). As the values of *I* ^2^ were greater than 50%, subgroup analyses were adopted according to stratification on gender of animals and the model construction. Effect size was greater in models of male rats than in male and female mixed models (Figure 6(a)) and was greater in the intragastric administration models than in intraperitoneal injection models (Figure 6(b)). One study [20] also reported the significant effects of ACRRR for reducing NFS according to eight-point criterion compared with control group (*P* < 0.01), but it did not provide primary data and failed for pool analysis.

### 3.5. Assessment of Publication Bias

**Figure 7:**
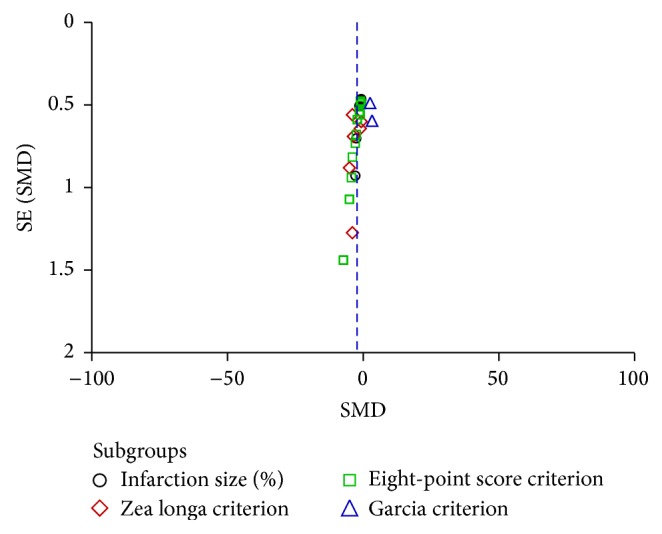
The funnel plot of assessing publication bias.

The funnel plot revealed a roughly symmetrical distribution of studies around the line of identity, indicating no obvious publication bias existed in this review (Figure 7).

## 4. Discussion

### 4.1. Summary of Evidences

This is the first preclinical systematic review evaluating the ACRRR for animal model of ischemic stroke with NFS and infarct size as the outcome measures. Twenty studies, involving a total of 577 experimental subjects, were identified. The quality of studies included in systematic review was generally low. The present study demonstrated that the ACRRR substantially reduced infarct size and improved NFS in animal models experiments of focal cerebral ischemia.

### 4.2. Methodological Considerations

This systematic review is subject to possible methodological weaknesses. First, our analysis can only include available data, and negative studies are often not published and obtained. Thus, the analysis may overestimate effect size. Second, we cannot rule out the possibility of missing relevant studies because our search strategy used only English and Chinese databases, which may lead to certain degree of selective bias. Third, the analysis rested with inherent limitations in the primary studies. Methodological quality of animal experiments is a significant concern because studies that report items such as blinding of outcomes and randomization are less prone to bias than are more rigorous studies [40]. Only 1 study [16] mentioned masked assessment of outcome, which may result in performance bias and detection bias. An adequate sample size is crucial to the design of randomized controlled trials [41]. Only six studies described a sample size calculation [16, 17, 20, 22, 26, 32]. Ischemic stroke generally occurs in elderly patients with associated medical problems such as hypertension and hyperglycemia. However, none of the studies investigated stroke in models with comorbidities such as diabetes, hypertension, or aged animals. Some anesthetic agents, including ketamine, have significant intrinsic neuroprotective activity [34], and experiments using these anesthetics may overestimate effect size, but no report of anesthetics in the 12 out of 20 studies.

### 4.3. Possible Neuroprotective Mechanism

The possible mechanisms, especially neuroprotective mechanism against cerebral ischemic injury, are summarized as follows: (i) rhubarb aglycone can reduce thrombosis, blood coagulation, and the aggregation and adhesion of platelet, decrease expression of fibrinogen, downregulate levels of tumor necrosis factor-*α* (TNF-*α*), interleukin-1*β* (IL-1*β*), and vascular cell adhesion molecule (VCAM-1), and upregulate transforming growth factor beta (TGF-*β*) in brain tissues [16, 20]; (ii) rhubarb glycosides can reduce the level of TNF-*α* and IL-1*β*, extracellular Ca^2+^ influx, and malate dehydrogenase (MDH) contents and increase superoxide dismutase (SOD) activity in brain tissue of MCAO rats [21, 22]; (iii) chrysophanol can reduce TNF-*α* level in mouse brain [24] and inhibit the NACHT domain-, leucine-rich repeat-, and pyrin domain-containing protein 3 (NALP3) inflammasome activation and it ameliorates cerebral ischemia/reperfusion in mice [33]; (iv) chrysophanol liposome has beneficial effects on neurobehavioral score and hippocampal pathological damage via increasing B-cell lymphoma-2 (Bcl-2) expression and reducing caspase-3 and Bax level in ischemic mice [32]; (v) emodin can reduce inflammatory cascade and increase TGF-*β* level [28] and inhibit the activation of caspase-3 in the cerebral ischemic model of SD rats [25]; (vi) aloe-emodin can provide neuroprotection against cerebral ischemic injury of SD rats by reduction of TNF-*α* level [24, 27]; (vii) physcion can enhance ischemic tolerance induced by brain ischemic preconditioning through decreasing IL-1*β*, TNF-*α*, ICAM-1, and caspase-3 expression in MCAO rats [42]; (viii) rhein has neuroprotective effects through reduction of level of nitric oxide (NO) and TNF-*α* in ischemic brain tissue of mice [43]. Thus, ACRRR have been demonstrated to be beneficial effects on multiaspects of the pathophysiology of stroke.

### 4.4. Implication for Further Practices and Studies

Although the relationship between study quality and the estimate of size of effect is not yet conclusive [44], some previous studies suggested that the quality of the research design is an important factor affecting the observed size of effect [34, 45]. On the practice level, we recommended the principles of randomization to treatment group, performance of surgery blinded to treatment allocation, blinded assessment of outcome, minimization of use of anesthetics with intrinsic neuroprotective activity, increased use of hypertensive, diabetic, and aged animals, and full reporting of potential conflicts of interests. In particular, ACRRR should be tested in aged, hyperpietic, and diabetic animals in future stroke studies because a metaepidemiologic approach by Crossley et al. [46] indicated that studies using healthy animals may overestimate the effectiveness of an intervention. On the study side, the relationship between study quality and the estimate of size of effect is an important area for future research.

It is worth noting that the neuroprotective activity of ACRRR for acute ischemic stroke may identify an area that other chemical compounds of RRR possess this activity. Second, which type of ACRRR possesses better neuroprotective function needs to be further clarified. Third, future studies of neuroprotective agents need to be tested in combination with different types of ACRRR to reduce the cellular effects of acute ischemia and to restore perfusion. Fourth, most of the studies in this field are explanatory on the therapeutic potential of ACRRR with little explanation of mechanism of action, especially on the causal relationship of the molecular or biological changes induced by ACRRR on therapeutic action. Thus, whether the neuroprotective effects of different types of ACRRR in acute ischemic stroke may have same or different molecular and biological mechanisms is worthy of further exploration. Fifth, further experimental studies with delayed ACRRR administration are required in order to assess when the optimum time window closes and to determine the time of administration under which maximum efficacy can be achieved.

## 5. Conclusion

The ACRRR can improve NFS and infarct size and exert potential neuroprotective effect for experimental ischemic stroke. However, these apparently positive findings should be interpreted with caution because of the methodological flaws. Future research should examine the presence of possible experimental bias and clinical trials of ACRRR are needed.

## Figures and Tables

**Table 1:** Study characteristics of included studies.

Study	Species (*n*)	Weight	Random method	Stroke model	Rhubarb effective component	Method of administration		The method to execute the animal	Outcome measure (experimental/control)	Intergroup differences
						Experimental group	Control group			
Li et al. 2005 [16]	Male, SD rats (10/10)	300 ± 50 g	Random digits table	Permanent MCAO	Rhubarb aglycone	4 d before occlusion; i.g; 103.68 mg/kg, daily	4 d before occlusion; i.g; same volume of normal saline, daily	Not mentioned	(1) Neurobehavioral score (2) Infarction size	(1) *P* < 0.05 (2) *P* < 0.05

Li et al. 2011 [17]	Male and female, SD rats (8/8)	300 ± 50 g	Not mentioned	Embolic MCAO	Rhubarb aglycone	4 h before occlusion; i.g; 12.96 mg/kg, daily	4 h before occlusion; i.g; same volume of normal saline, daily	Not mentioned	(1) Neurobehavioral score (2) Infarction size	(1) *P* < 0.01 (2) *P* < 0.01

Li et al. 2005 [18]	Male, SD rats (8/10)	300 ± 50 g	Random digits table	Permanent MCAO	Rhubarb aglycone	5 d before occlusion; i.g; 25.92 mg/kg, daily	MCAO without any intervention	Not mentioned	Neurobehavioral score	*P* < 0.01

Li et al. 2007 [19]	Male and female, SD rats (10/10)	300 ± 50 g	Random digits table	Permanent MCAO	Rhubarb aglycone	3 d before occlusion; i.p; 103.68 mg/kg, daily	3 d before occlusion; i.p; same volume of normal saline, daily	Not mentioned	(1) Neurobehavioral score (2) Infarction size	(1) *P* < 0.05 (2) Not found

Li et al. 2005 [20]	Male and female, SD rats (6/6)	300 ± 50 g	Random digits table	Permanent MCAO	Rhubarb aglycone	3 d before occlusion; i.p; 103.68 mg/kg, daily	3 d before occlusion; i.p; same volume of normal saline, daily	Not mentioned	(1) Neurobehavioral score (2) Infarction size	(1) *P* < 0.01 (2) *P* < 0.01

Liu et al. 2005 [21]	Male, SD rats (8/10; 8/10)	300 ± 50 g	Not mentioned	Permanent MCAO	Rhubarb aglycone	5 d before occlusion; i.g; 25.92 mg/kg, daily	MCAO without any intervention	Not mentioned	Neurobehavioral score	*P* < 0.01
					Rhubarb glycosides	5 d before occlusion; i.g; 174.96 mg/kg, daily			Neurobehavioral score	*P* < 0.01

Li et al. 2004 [22]	Male, SD rats (8/10; 8/10)	300 ± 20 g	Random digits table	Permanent MCAO	Rhubarb aglycone	5 d before occlusion; i.g; 25.92 mg/kg, daily	MCAO without any intervention	Not mentioned	Neurobehavioral score	*P* < 0.01
					Rhubarb glycosides	5 d before occlusion; i.g; 174.96 mg/kg, daily			Neurobehavioral score	*P* < 0.01

Li et al. 2004 [23]	Male, SD rats (8/10; 8/10)	300 ± 50 g	Random digits table	Permanent MCAO	Rhubarb glycosides	3 d before occlusion; i.p; 174.96 mg/kg, daily	MCAO without any intervention	Not mentioned	Neurobehavioral score	*P* < 0.01
					Rhubarb aglycone	3 d before occlusion; i.p; 25.92 mg/kg, daily			Neurobehavioral score	*P* < 0.01

Liu et al. 2004 [24]	Male, SD rats (10/9; 9/9; 10/9; 8/9; 9/9; 9/9)	300 ± 50 g	Random digits table	Permanent MCAO	Rhubarb aglycone	3 d before occlusion; i.p; 25.92 mg/kg, daily	MCAO without any intervention	Not mentioned	(1) Neurobehavioral score (2) Infarction size	(1) *P* < 0.05 (2) *P* < 0.05
					Emodin	3 d before occlusion; i.p; 1.404 mg/kg, daily			(1) Neurobehavioral score (2) Infarction size	(1) *P* < 0.01 (2) *P* < 0.05
					Aloe-emodin	3 d before occlusion; i.p; 0.648 mg/kg, daily			(1) Neurobehavioral score (2) Infarction size	(1) *P* < 0.05 (2) Not found
					Physcion	3 d before occlusion; i.p; 1.08 mg/kg, daily			(1) Neurobehavioral score (2) Infarction size	(1) Not found (2) Not found
					Rhein	3 d before occlusion; i.p; 3.46 mg/kg, daily			(1) Neurobehavioral score (2) Infarction size	(1) Not found (2) *P* < 0.05
					Chrysophanol	3 d before occlusion; i.p; 7.88 mg/kg, daily			(1) Neurobehavioral score (2) Infarction size	(1) *P* < 0.05 (2) *P* < 0.05

Tan et al. 2010 [25]	Male, SD rats (5/5)	250–320 g	Not mentioned	Temporary MCAO	Emodin	30 min before occlusion; i.p; 25 mg/kg	MCAO without any intervention	Anesthetized	(1) Neurobehavioral score (2) Infarction size	(1) *P* < 0.01 (2) *P* < 0.01

Wu et al. 2009 [26]	Male, SD rats (6/6)	270–300 g	Random digits table	Temporary MCAO	Emodin	3 d before occlusion; i.p; 25 mg/kg, daily	3 d before occlusion; i.p; same volume of normal saline, daily	Not mentioned	Neurobehavioral score	*P* < 0.05

Wang et al. 2005 [27]	Male and female, SD rats (10/10; 10/10)	300 ± 50 g	Random digits table	Permanent MCAO	Emodin	3 d before occlusion; i.p; 5.616 mg/kg, daily	3 d before occlusion; i.p; same volume of normal saline, daily	Not mentioned	(1) Neurobehavioral score (2) Infarction size	(1) *P* < 0.01 (2) *P* < 0.05
					Aloe-emodin	3 d before occlusion; i.p; 0.162 mg/kg, daily			(1) Neurobehavioral score (2) Infarction size	(1) *P* < 0.01 (2) *P* < 0.01

Li et al. 2005 [28]	Male and female, SD rats (10/10)	300 ± 50 g	Random digits table	Permanent MCAO	Emodin	3 d before occlusion; i.p; 5.616 mg/kg, daily	3 d before occlusion; i.p; same volume of normal saline, daily	Not mentioned	(1) Neurobehavioral score (2) Infarction size	(1) *P* < 0.01 (2) *P* < 0.05

Chen et al. 2006 [29]	Male, SD rats (12/12)	250–320 g	Not mentioned	Temporary MCAO	Physcion	3 d before occlusion; i.g; 40 mg/kg, daily	3 d before occlusion; i.p; same volume of normal saline, daily	Anesthetized	(1) Neurobehavioral score (2) Infarction volume	(1) *P* < 0.01 (2) *P* < 0.01

Mei et al. 2009 [30]	Male, SD rats (12/12)	250–320 g	Not mentioned	Temporary MCAO	Physcion	3 d before occlusion; i.g; 60 mg/kg, daily	MCAO without any intervention	Not mentioned	(1) Neurobehavioral score (2) Infarction volume	(1) *P* < 0.01 (2) *P* < 0.01

Chen et al. 2007 [31]	Male, SD rats (10/10)	250–320 g	Not mentioned	Temporary MCAO	Physcion	3 d before occlusion; i.g; 40 mg/kg, daily	MCAO without any intervention	Not mentioned	(1) Neurobehavioral score (2) Infarction volume	(1) *P* < 0.01 (2) *P* < 0.01

Song et al. 2011 [32]	Male, Kunming mice (15/15; 15/15)	28.0 ± 0.9 g	Not mentioned	Temporarily obstructing bilateral common carotid arteries (Himori method)	Chrysophanol	14 d after occlusion; i.p; 10.0 mg/kg, daily	14 d before occlusion; i.p; same volume of normal saline, daily	Anesthetized	Neurobehavioral score	Not found
					Chrysophanol liposome	14 d after occlusion; i.p; 10.0 mg/kg, daily			Neurobehavioral score	Not found

Zhang et al. 2014 [33]	Male, CD1 mice (6/6)	25∼30 g	Not mentioned	Temporary MCAO	Chrysophanol	30 minutes before occlusion; i.p; 10.0 mg/kg, daily	30 minutes before occlusion; i.p; same volume of normal saline, daily	Anesthetized	(1) Neurobehavioral score (2) Infarction size	(1) *P* < 0.05 (2) *P* < 0.05

Chen et al. 2015 [14]	Male, SD rats (20/20)		Random digits table	Permanent MCAO	Physcion	3 d before occlusion; i.g; 40 mg/kg, daily	3 d before occlusion; i.p; same volume of normal saline, daily	Not mentioned	(1) Neurobehavioral score (2) Infarction volume	(1) *P* < 0.01 (2) *P* < 0.01

Guan et al. 2014 [15]	Male, Wistar rats (6/6)	280 ± 20 g	Not mentioned	Permanent MCAO	Rhubarb aglycone	4 d before occlusion; i.g; aloe-emodin 50 mg/kg, rhein 76 mg/kg, emodin 38 mg/kg, chrysophanol 105 mg/kg, physcion 68 mg/kg, daily	4 d before occlusion; i.g; same volume of 0.5% CMC-Na suspension	Not mentioned	(1) Neurobehavioral score (2) Infarction size	(1) *P* < 0.05 (2) *P* < 0.01

Note: rhubarb aglycone referred to the five components including aloe-emodin, rhein, emodin, chrysophanol, and physcion. Rhubarb glycosides referred to anthraquinone glycosides and double anthrone glycoside. IL-1*β*: interleukin-1*β*; MDH: malate dehydrogenase; MCAO: middle carotid artery occlusion; NALP3: NACHT domain-, leucine-rich repeat-, and pyrin domain-containing protein 3; NF-KB: nuclear factor-kappa B; SOD: superoxide dismutase; TGF-*β*: transforming growth factor beta; TNF-*α*: tumor necrosis factor-*α*; VCAM-1: vascular cell adhesion molecule.

**Table 2:** Quality characteristics of included studies.

Study	A	B	C	D	E	F	G	H	I	J	Score
Li et al. 2005 [16]	+	−	+	−	+	+	−	−	?	−	4
Li et al. 2011 [17]	+	−	+	−	−	?	−	+	?	−	3
Li et al. 2005 [18]	+	−	+	−	−	?	−	−	?	−	2
Li et al. 2007 [19]	+	+	+	−	−	?	−	−	?	−	3
Li et al. 2005 [20]	+	+	+	−	−	?	−	+	?	−	4
Liu et al. 2005 [21]	+	−	+	−	−	?	−	−	?	−	2
Li et al. 2004 [22]	+	+	+	−	−	?	−	+	?	−	4
Li et al. 2004 [23]	+	+	+	−	−	?	−	−	?	−	3
Liu et al. 2004 [24]	+	−	+	−	−	?	−	−	?	−	2
Tan et al. 2010 [25]	+	−	+	−	−	+	?	−	?	−	3
Wu et al. 2009 [26]	+	+	+	−	−	+	?	+	?	−	5
Wang et al. 2005 [27]	+	+	+	−	−	?	−	−	?	−	3
Li et al. 2005 [28]	+	+	+	−	−	?	−	−	?	−	3
Chen et al. 2006 [29]	+	+	+	−	−	+	?	−	?	−	4
Mei et al. 2009 [30]	+	−	+	−	−	+	?	−	?	−	3
Chen et al. 2007 [31]	+	−	+	−	−	?	?	−	?	−	2
Song et al. 2011 [32]	+	+	+	−	−	+	?	+	?	−	5
Zhang et al. 2014 [33]	+	+	+	−	−	+	?	−	+	+	6
Chen et al. 2015 [14]	+	−	+	−	−	+	−	−	?	−	3
Guan et al. 2014 [15]	+	+	+	−	−	+	−	−	?	−	4

Note: A: publication in a peer-reviewed journal, B: statement of temperature control, C: random allocation to groups, D: blinded induction of ischemia, E: blinded assessment of outcome, F: use of anaesthetic without significant intrinsic neuroprotective activity, G: appropriate animal model (aged, diabetic, or hypertensive), H: sample size calculation, I: compliance with animal welfare regulations, and J: statement of potential conflict of interests. +: yes, −: no, and ?: unclear.
